# Use of monofilament sutures and triclosan coating to protect against surgical site infections in spinal surgery: a laboratory-based study

**DOI:** 10.1007/s00590-023-03534-w

**Published:** 2023-03-31

**Authors:** H. M. T. Fawi, P. Papastergiou, F. Khan, A. Hart, N. P. Coleman

**Affiliations:** 1 Trauma and Orthopaedics Department, Queen Elizabeth Hospital NHS Trust, Kings Lynn, UK; 2 School of Public Health, Imperial College London, London, UK; 3 Microbiology Department, Limassol General Hospital, Kato Polemidia, Cyprus; 4 Microbiology Department, Norfolk & Norwich University Hospital NHS Trust, Norwich, UK; 5 School of Clinical Medicine, University of Cambridge, Cambridge, UK

**Keywords:** Infections, Sutures, Triclosan, Post-operative care, Surgical site infections, Spinal surgery

## Abstract

**Purpose:**

We investigated bacterial propagation through multifilament, monofilament sutures and whether sutures coated with triclosan would exhibit a different phenomenon.

**Methods:**

One centimetre (cm) wide trenches were cut in the middle of Columbia blood Agar plates. We tested a 6 cm length of two Triclosan-coated (PDS plus®, Vicryl plus®) and two uncoated (PDS ®, Vicryl ®) sutures. Each suture was inoculated with a bacterial suspension containing methicillin-sensitive *Staphylococcus aureus* (MSSA), Escherichia coli (*E. coli*), Staphylococcus epidermidis, methicillin-resistant Staphylococcus aureus (MRSA) at one end of each suture. The plates were incubated at 36C for 48 h, followed by room temperature for a further 5 days. We established bacterial propagation by observing for any bacterial growth on the Agar on the opposite side of the trench.

**Results:**

Bacterial propagation was observed on the opposite side of the trench with both suture types, monofilament PDS and multifilament Vicryl, when tested with the motile bacterium (*E. coli*). Propagation was not observed on the other side of the trench with the monofilament PDS suture following incubation with MSSA and *S. epidermidis*, and in 66% of MRSA. With multifilament suture Vicryl, propagation was observed on the other side of the trench in 90% (MSSA), 80% (*S. epidermidis*), and 100% (MRSA) of plates tested. No bacterial propagation was observed in any of the triclosan-coated sutures (monofilament or multifilament).

**Conclusions:**

Monofilament sutures are associated in vitro with less bacterial propagation along their course when compared to multifilament sutures. Inhibition in both sutures can be further enhanced with a triclosan coating.

## Introduction

Postoperative surgical site infection (SSI) following spinal surgery is a serious complication, with incidence rates reported to be up to 16% [1–4]. SSIs are reported to be the third most common complication of spinal surgery [5–7]. They are associated with significant morbidity and have a heavy economic burden [8]. In the USA, the direct and indirect health care costs associated with SSIs were reported to reach up to 10 billion USD, with up to 8000 death per year [9].

The Gram-positive bacteria (*Staphylococci*, *Streptococci*, *Enterococci*, *Propionibacterium*) are the most common organisms responsible for spinal SSI. Among the Gram positive bacteria, *Staphylococcus aureus* and *Staphylococcus epidermis* account for the majority of isolated organisms. The less common gram-negative bacteria, such as* Pseudomonas aeruginosa*, *Escherichia coli*, and Proteus species, may account for up to one-third of cases of spinal SSI [10–14].

Researchers have looked at modifiable risk factors with the aim of improving rates of SSI post-spinal surgery [15]. For example, Masaki et al. assessed the effect of triclosan-coated sutures on wound infections following spinal surgery, reporting that their use may reduce the incidence of postoperative SSI [16]. Furthermore, suture material can change the susceptibility to bacterial infections in surgical wounds [17]. Suture products have evolved over the years from natural to synthetic, in monofilament and multifilament forms with the synthetic multifilament Vicryl (Ethicon-Johnson & Johnson Medical Limited, Ohio, USA), ubiquitously used across the surgical specialities [18]. Both types (monofilament, multifilament) of sutures have their advances and disadvantages. Monofilament sutures require careful handling and tying. There is a risk of crushing or crimping of the suture can nick or weaken it which can lead to undesirable and suture failure. Multifilament sutures generally have greater tensile strength and better pliability and have relative greater flexibility in comparison with monofilament suture material. In addition, multifilament sutures have superior handling properties. In general, which suture is used depends on the surgeon’s preference.

Recent suture research has focused on triclosan-coated multifilament sutures (Ethicon, Somerville, New Jersey) [16, 18, 19]. Several high-profile organisations, such as the World Health Organisation (WHO), and National Institute for Health and Care Excellence (NICE), have been advocating the use of triclosan-coated sutures [20, 21]. Noticeably, the published literature about these sutures varies in their reported outcomes. Some recommend their use whilst other studies showed no statistical significance associated with their use [16, 18, 19].

To date, only one historic paper published in 1977 has quantitatively assessed the phenomena of bacterial transport through different suture materials [22]. Their experiments were conducted both in laboratory and rat models, using monofilaments and multifilament sutures. The researchers concluded that the tested immobile bacteria, Staphylococcus aureus, were transported inside multifilament materials. Similar results were obtained in their in vivo arm of the study in rats’ muscles. Bacterial transport was not observed through the monofilament sutures. The sutures used during the 1977 study are not currently in use.

Our aim was to investigate bacterial propagation through monofilament, multifilament sutures, and those coated with triclosan, using contemporary sutures in an in vitro, laboratory-based study. We used some of the commonly encountered bacteria associated with spinal surgical site infections (SSI) [23–26]. We hypothesised that synthetic monofilament sutures would be associated with less risk of bacterial propagation through suture strands in comparison with synthetic multifilament sutures. In addition, triclosan coating might have a synergistic effect to reduce bacterial propagation further.

## Materials and methods

### Suture material

The following suture materials were tested: PDS (Polydioxanone) size 1, PDS Plus size 1; Vicryl (Polyglactin 910) size 2.0, and Vicryl Plus size 2.0 (Ethicon-Johnson & Johnson Medical Limited).

PDS is a synthetic absorbable monofilament suture. It elicits minimal tissue reaction. It retains 80% of its strength by 14 days, 70% by 28 days, and 60% by 42 days. Absorption is essentially complete in 182–238 days [27].

Vicryl is a synthetic absorbable multifilament that elicits minimal acute inflammatory reaction. It retains 75% of its tensile strength at two weeks post-implantation, and 50% at three weeks. All the original tensile strength is lost between 4–5 weeks post-implantation. Absorption is essentially complete by 56–70 days [27].

The PDS Plus and Vicryl Plus sutures contained antibacterial Triclosan in the form of Irgacare® MP ≤ 2360 μg/m, and ≤ 472 μg/m, respectively [28].

Triclosan is an antimicrobial agent that has a broad range of activity against many Gram-positive, Gram-negative bacteria, and some fungi. Triclosan is bacteriostatic at low concentrations, but higher levels are bactericidal [29].

### Methods

Petri dishes containing Columbia blood Agar (Sheep blood 5%) from Liofilchem® were used. A one centimetre (cm) wide trench was cut with a sterile scalpel in the middle of the agar plate. This trench created a complete gap in the agar plate to prevent bacterial spread from one side of the trench to the other directly through the agar.

A 6 cm length of each type of suture was placed on the Petri dish across the trench. A saline solution containing 0.5 McFarland bacterial suspensions was prepared. The 0.5 McFarland turbidity standard provides an optical density comparable to the density of a bacterial suspension with a 1.5 × 10^8^ colony forming units (CFU/ml). The bacterial suspension used included one of the following four organisms: methicillin-sensitive *Staphylococcus aureus* (MSSA), (American Type Culture Collection (ATCC) Virginia, USA) ATCC29213; *Escherichia coli* ATCC25922; *Staphylococcus epidermidis* ATCC12228; Methicillin-resistant *Staphylococcus aureus* (MRSA) ATCC213300. In total, 80μL of a single bacterial suspension was inoculated to one end of each suture, in instalments of 20 μL every 30 min. Inoculation of the bacterial suspension was performed in a biosafety cabinet. The plates were incubated at 36 °C for 48 h and subsequently were kept at room temperature for a further five days.

We observed macroscopically for any presence of bacterial growth on the agar on the opposite side of the trench to establish any tracking/transfer of the organism. For sutures coated with triclosan, we also observed for the presence of a bacterial growth inhibition zone. We continued to observe whether the zone of growth inhibition was sustained until day seven.

### Statistical analysis

Undertaken using Fisher’s exact test for categorical data. Statistical significance was set at *p* ≤ 0.05, and statistical analyses were calculated using IBM SPSS Statistics, Version 26.

## Results

### Monofilament PDS in comparison with multifilament Vicryl sutures without triclosan

Propagation was observed onto the other side of the trench with both suture types with *E. coli* (100%). This was not observed on the other side of the trench with the majority of monofilament (PDS) sutures following incubation with MSSA, *S. epidermidis*, and MRSA.

Propagation was observed on the other side of the trench in the vast majority with multifilament Vicryl sutures following incubation with MSSA, *S. epidermidis*, and MRSA (Table 1).

**Table 1:** PDS (monofilament) vs Vicryl (multifilament)

Organism	Total no of sutures tested	Total no of PDS with observed propagation of bacteria (Day 7) *n* (%)	Total no of *Vicryl* with observed propagation of bacteria (Day 7) *n* (%)	*p* value
*E.coli* ATCC25922	10	10 (100%)	10 (100%)	1.000
MSSA ATCC29213	10	0 (0%)	9 (90%)	< 0.001
*S. epidermidis* ATCC12228	10	0 (0%)	8 (80%)	< 0.001
MRSA ATCC213300	6	2 (66%)	6 (100%)	0.06

### Triclosan-coated sutures (PDS plus and Vicryl plus)

**Fig. 1:**
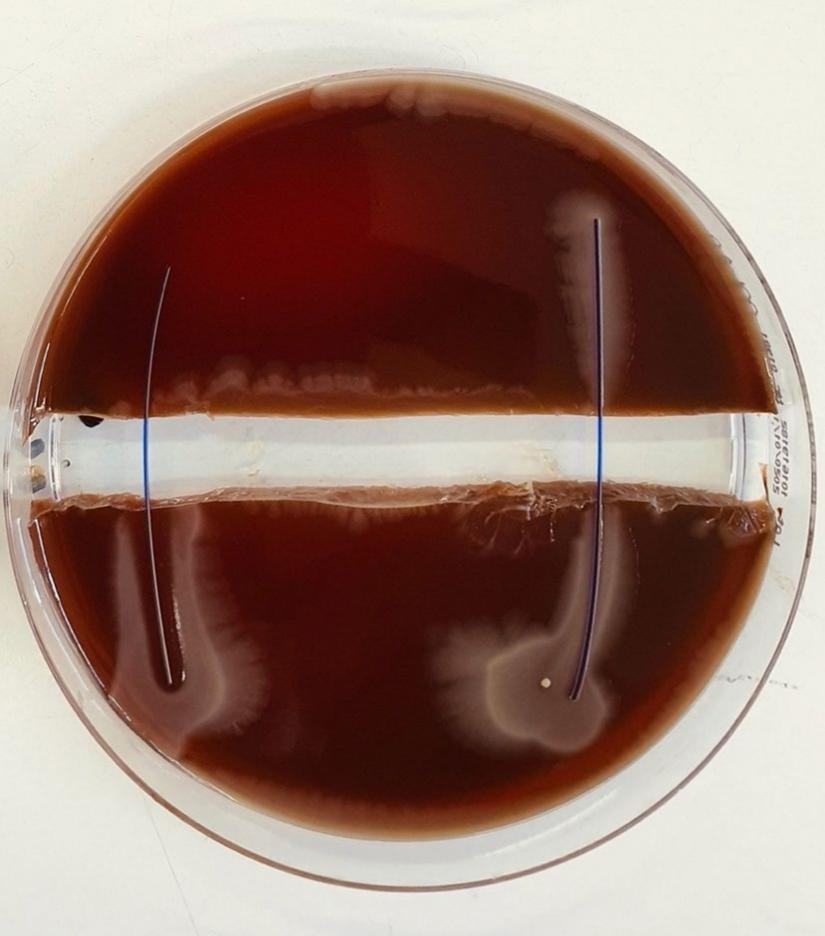
PDS (RIGHT) vs. PDS Plus (LEFT) were tested with E. coli ATCC25922. Demonstrates propagation over PDS across the trench, whilst PDS Plus shows a well-marked zone of inhibition, with no bacterial propagation

**Fig. 2:**
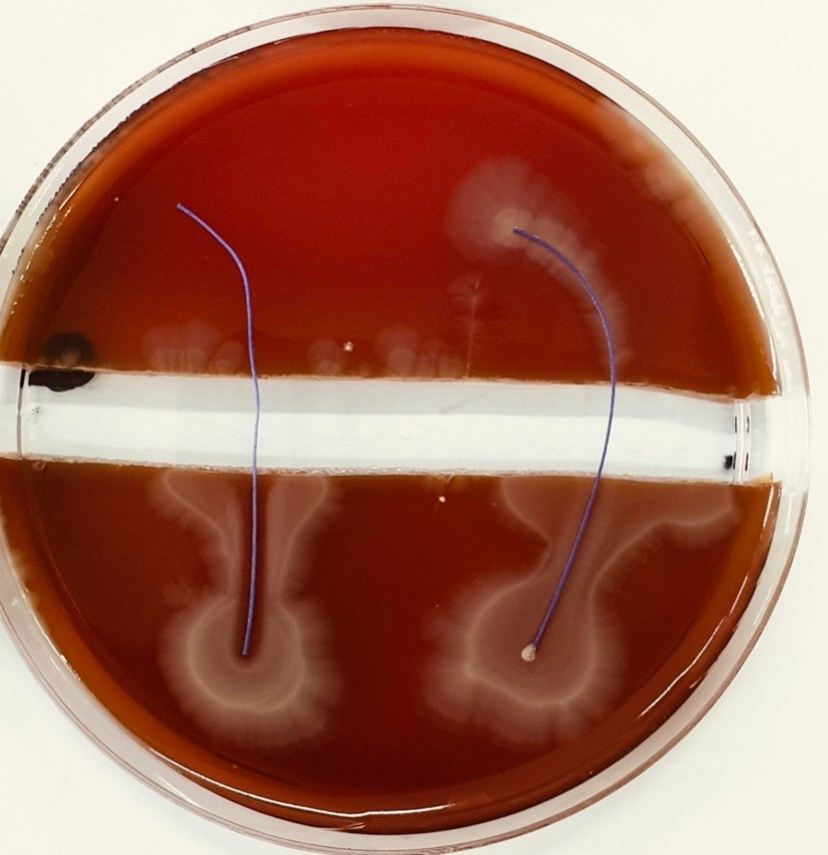
Vicryl (RIGHT) vs. Vicryl Plus (LEFT) were tested with *E. coli* ATCC25922. Demonstrates propagation over Vicryl across the trench. Vicryl Plus shows a well-marked zone of inhibition with no bacterial propagation.

**Fig. 3:**
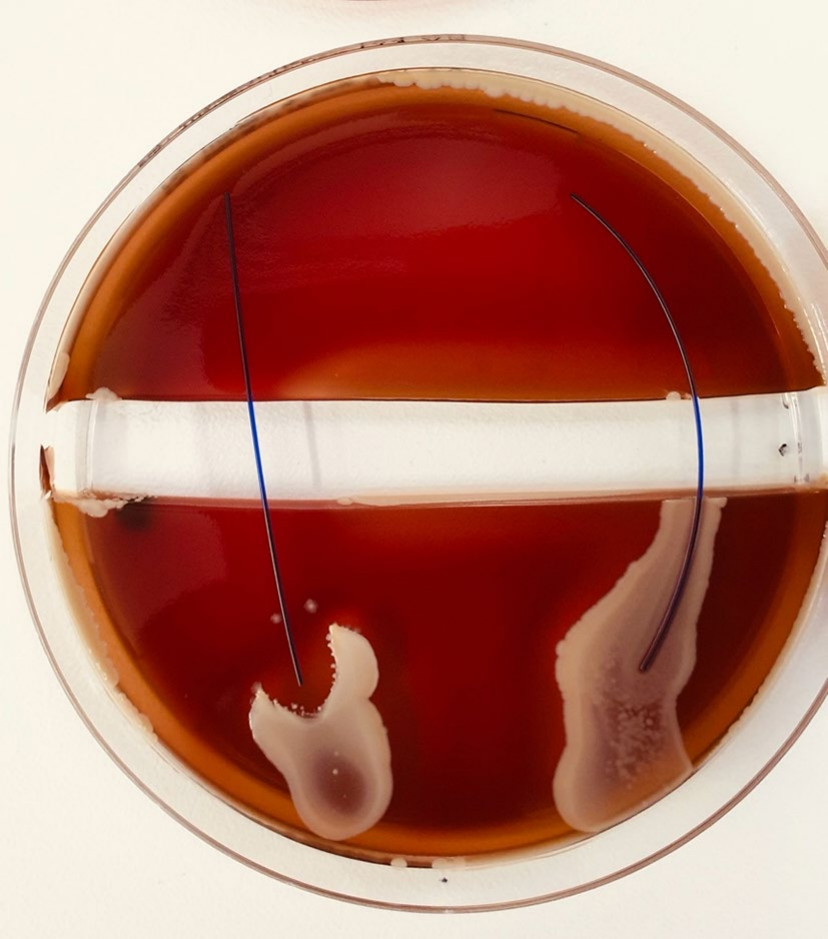
PDS (RIGHT) vs. PDS Plus (LEFT) were tested with MSSA ATCC29213. Demonstrates no propagation over the trench, on both sutures. A clear zone of inhibition is observable around the PDS Plus

**Fig. 4:**
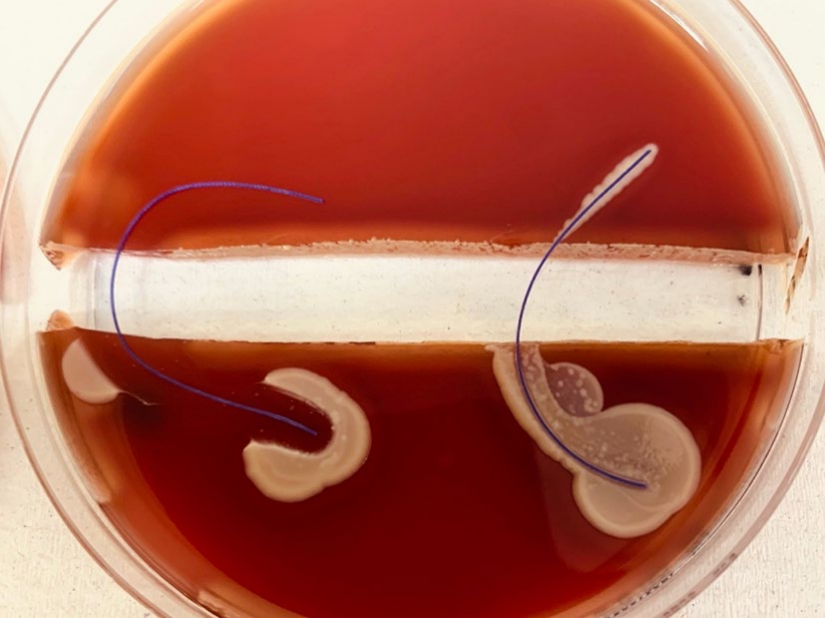
Vicryl (RIGHT) vs. Vicryl Plus (LEFT) were tested with MSSA ATCC29213. Demonstrates propagation over Vicryl across the trench. Vicryl Plus shows a well-marked zone of inhibition with no bacterial propagation

**Fig. 5:**
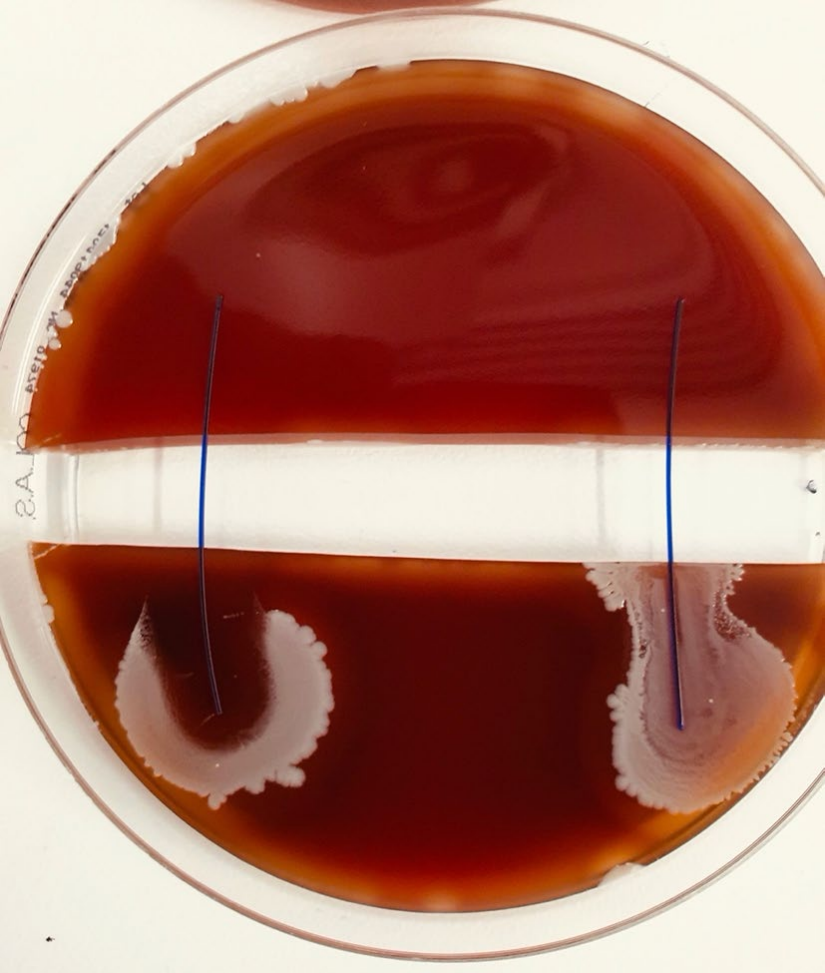
PDS (RIGHT) vs. PDS Plus (LEFT) were tested with S. epidermidis ATCC12228. Demonstrates no propagation over the trench, on both sutures. In addition, a clear zone of inhibition is visible around the PDS Plus

**Fig. 6:**
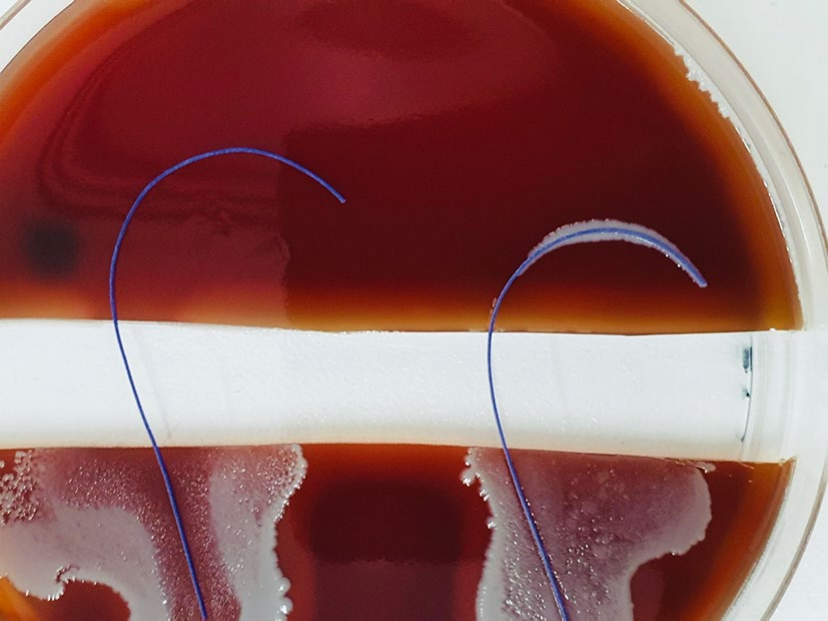
Vicryl (RIGHT) vs. Vicryl Plus (LEFT) were tested with S. epidermidis ATCC12228. Demonstrates propagation over Vicryl across the trench. Vicryl Plus showed a zone of inhibition, with no bacterial propagation

**Fig. 7:**
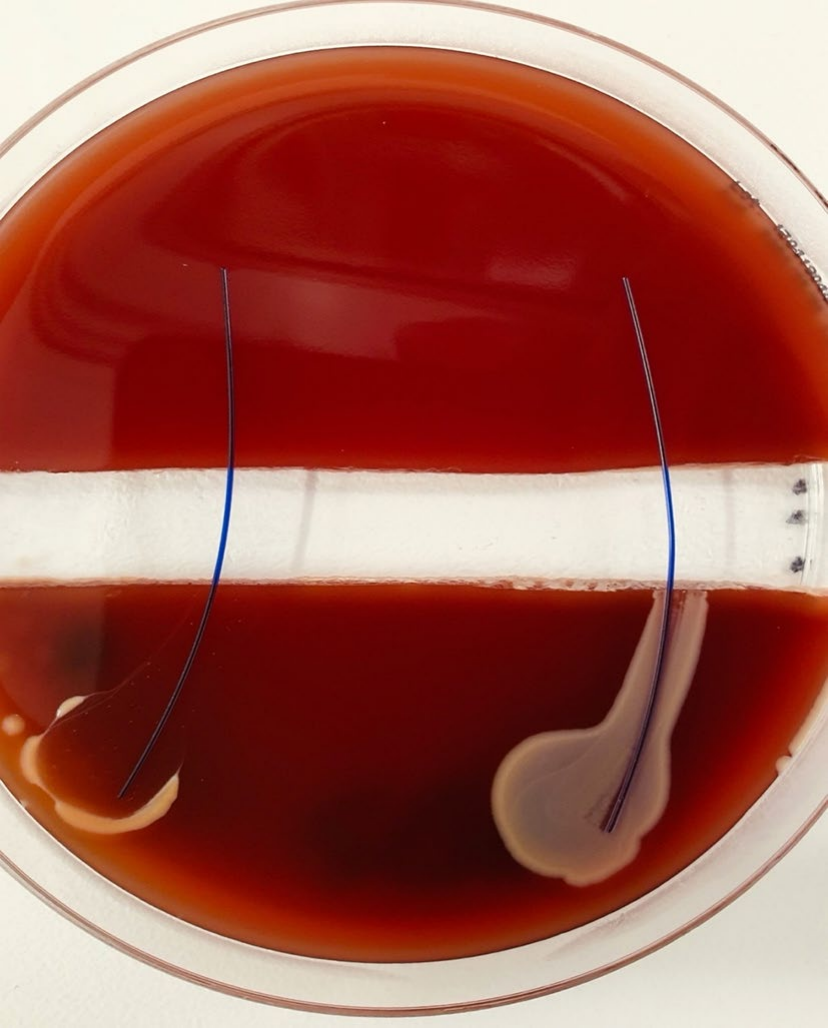
PDS (RIGHT) vs. PDS Plus (LEFT) were tested with MRSA ATCC213300. Demonstrates no propagation over the trench, on both sutures. A clear zone of inhibition is observable around the PDS Plus

**Fig. 8:**
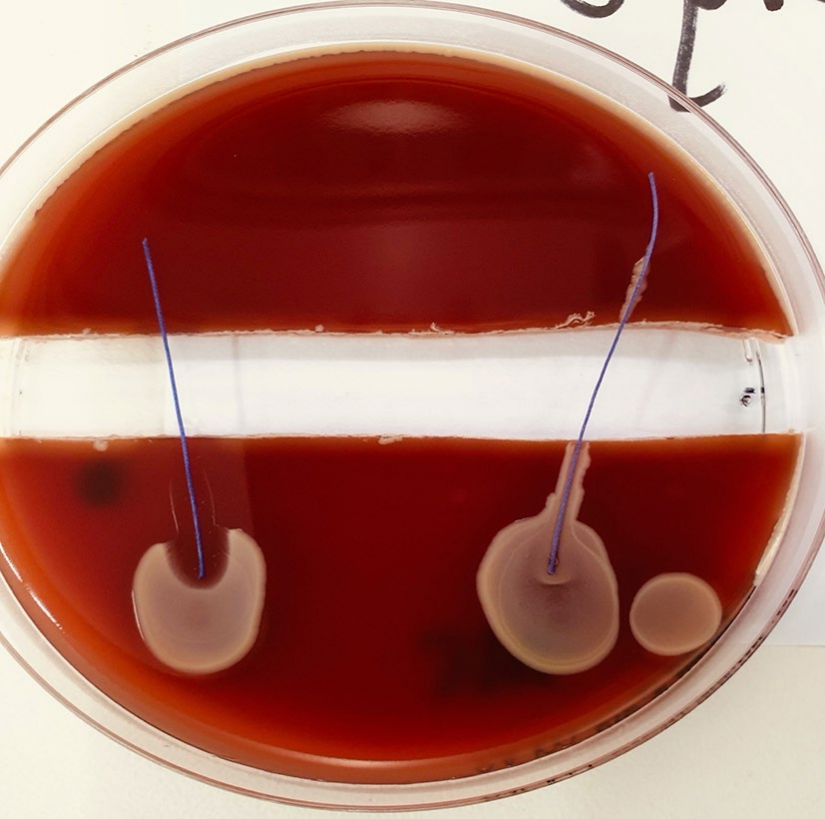
Vicryl (RIGHT) vs. Vicryl Plus (LEFT) were tested with MRSA ATCC213300. Vicryl demonstrates propagation across the trench, whilst Vicryl Plus showed a well-marked zone of inhibition, with no bacterial propagation

Sutures with triclosan coating showed broad bacteria inhibition zones around them (Figs. 1, 2, 3, 4, 5, 6, 7, 8). Propagation onto the other side of the trench was not observed with the tested organisms (*E. coli*, MSSA, MRSA, and *S. epidermidis*). The results are presented in Tables 2 and 3. The inhibition of bacterial growth was retained up to seven days of incubation. No statistically significant difference was found between the triclosan-coated PDS Plus and the Vicryl Plus sutures.

**Table 2:** PDS Plus (triclosan coated) vs PDS

Organism	Total no of sutures tested	Total no of PDS Plus with observed propagation of bacteria (Day 7) *n* (%)	Total no of *PDS* with observed propagation of bacteria (Day 7) *n* (%)	*p* value
*E. coli* ATCC25922	10	0 (0%)	10 (100%)	< 0.001
MSSA ATCC29213	10	0 (0%)	0 (0%)	1.000
*S. epidermidis* ATCC12228	10	0 (0%)	0 (0%)	1.000
MRSA ATCC213300	6	0 (0%)	2 (33%)	0.45

**Table 3:** Vicryl Plus (triclosan coated) vs Vicryl

Organism	Total no of sutures tested	Total no of Vicryl Plus with observed propagation of bacteria (Day 7) *n* (%)	Total no of *Vicryl* with observed propagation of bacteria (Day 7) *n* (%)	*p* value
*E. coli* ATCC25922	10	0 (0%)	10 (100%)	< 0.001
MSSA ATCC29213	10	0 (0%)	9 (90%)	< 0.001
*S. epidermidis* ATCC12228	10	0 (0%)	8 (80%)	< 0.001
MRSA ATCC213300	6	0 (0%)	6 (100%)	< 0.001

## Discussion

To the best of our knowledge, this is the first study investigating bacterial propagation through monofilament PDS and multifilament Vicryl sutures, with and without triclosan coating. We used a variable selection of commonly encountered organisms in orthopaedics SSIs [23–26]. The tested non-motile bacteria were able to propagate through multifilament sutures to the other side of the trench. This was not observed in the majority of the tested monofilament sutures. The absence of propagation associated with the monofilament suture was not retained when motile bacteria were tested as both sutures showed propagation when *E.coli* was tested.

Our study is not without limitations. Confounding variables such as suture material, structure, and capillarity may influence the observed bacterial propagation differences between the PDS monofilament and Vicryl multifilament sutures [17]. The PDS monofilament sutures are made from the polyester (p-dioxanone). The Vicryl multifilament sutures are from a copolymer, Polyglactin 910 which is composed of 90% glycolide, 10% l-lactide and calcium stearate. Monofilament sutures are single stranded sutures, whereas multifilament sutures are made of several filaments twisted together. Multifilament sutures have narrow tubular inner spaces that can transport bacteria as they transport fluids, similar to a wick [30]. This is due to the capillary ascent phenomenon where fluids can rise in a narrow tube when submerged, as a result of the molecular activity between adjacent bodies [31].

The spread of flagellated bacteria on surfaces can involve millions of bacteria moving together in complex patterns, through collective motion. This includes rapid migration on surfaces in groups through phenomena such as swarming and near-surface swimming [32, 33]. Previous research has also suggested that bacteria can use sutures as a guide rail for movement [30]. Although our study was conducted in vitro and its clinical relevance may be limited, our findings are supported by previous research and animal studies, which suggest that the observed bacterial propagation on suture surfaces can also occur in vivo after spinal operations [22]. Studies on animal models demonstrated that barbed monofilament suture performed similarly to monofilament suture and better than braided suture in terms of bacterial adherence, biofilm formation, and tissue reactivity [34].

We found that neither motile nor non-motile bacteria were able to spread through triclosan-coated sutures. We observed a sustained zone of bacterial growth inhibition around the triclosan-coated sutures throughout the entire duration of our study (168 h) for all types of bacteria tested. The bacterial suspensions used in our study were at a concentration of 1.5 × 108 CFU/ml, as determined by a 0.5 McFarland standard for *E. coli*. Previous research has also shown that triclosan is able to inhibit bacterial adhesion to sutures for at least 96 h, which is consistent with our findings [35]. However, further studies with longer duration are needed to determine how long this inhibition is sustained.

Our study found that monofilament sutures have a reduced risk of bacterial propagation compared to multifilament sutures. However, adding a triclosan coating to the suture may provide additional protection, especially against motile Gram-negative bacteria, which can cause up to one-third of spinal SSIs [36]. However, it is important to note that triclosan is not effective against all types of bacteria and resistance in some organisms has been documented [29, 37, 38]. Therefore, using monofilament sutures coated with triclosan could provide additional protection against susceptible bacteria and act as a secondary defence mechanism against the spread of infection in case of a resistant bacteria.

Bacteria can colonise sutures as they are implanted, potentially developing a biofilm, a complex and well-structured aggregation of microorganisms of single or multiple species [39, 40]. Biofilm associated infection is one of the most common causes of failure of orthopaedics and Spinal operations [40]. While animal studies have demonstrated rapid (minutes to hours) biofilm formation, some studies suggest that biofilm formation may also not correlate with the onset of infection [40]. The biofilm formed in suture knots, for example, can lie dormant for years, further highlighting the need for longer-tern studies [41].

Analysis of biofilm formation and electronic microscopy were not implemented in this study, but may form essential aspects of further research to identify biofilm formation and microscopic bacterial propagation through sutures [42]. Future research to improve surgical sutures might benefit from incorporating the biophysical interactions between bacteria and surfaces and assessing whether micro-structuring sutures’ surfaces could mitigate bacterial propagation through them or over their surfaces [32]. Novel surgical sutures may benefit from structural properties that inhibit, and disturb the biofilm formation.

## Conclusion

In conclusion, our in vitro study demonstrated that the use of PDS monofilament sutures is associated with a lower likelihood of bacterial propagation along its course when compared to multifilament sutures. This suggests that monofilament PDS sutures may be a more suitable option for closing spinal wounds in order to reduce the risk of postoperative surgical site infections (SSIs). The use of triclosan-coated PDS monofilament sutures could further enhance the benefits of using this type of suture, assuming there are no contraindications [28]. While suture type is just one factor that influences surgical site infection, cumulatively, small improvement in reducing the risk of postoperative complications may lead to significant benefits in the success of spinal operations [43, 44].
